# Correlation between target motion and the dosimetric variance of breast and organ at risk during whole breast radiotherapy using 4DCT

**DOI:** 10.1186/1748-717X-8-111

**Published:** 2013-05-02

**Authors:** Wei Wang, Jian Bin Li, Hong Guang Hu, Feng Xiang Li, Min Xu, Tao Sun, Jie Lu

**Affiliations:** 1 Department of Radiation Oncology (Chest section), Shandong Cancer Hospital, Jinan, Shandong Province, 250117, P.R. China; 2 Department of Medical Physics, Shandong Cancer Hospital, Jinan, Shandong Province, 250117, P.R. China

## Abstract

**Purpose:**

The purpose of this study was to explore the correlation between the respiration-induced target motion and volume variation with the dosimetric variance on breast and organ at risk (OAR) during free breathing.

**Methods and materials:**

After breast-conserving surgery, seventeen patients underwent respiration-synchronized 4DCT simulation scans during free breathing. Treatment planning was constructed using the end inspiration scan, then copied and applied to the other phases and the dose distribution was calculated separately to evaluate the dose-volume histograms (DVH) parameters for the planning target volume (PTV), ipsilateral lung and heart.

**Results:**

During free breathing, the treated breast motion vector was 2.09 ± 0.74 mm, and the volume variation was 3.05 ± 0.94%. There was no correlation between the breast volume and target/OAR dosimetric variation (|r| = 0.39 ~ 0.48). In the anteroposterior, superoinferior and vector directions, breast movement correlated well with the mean PTV dose, conformal index, and the lung volume receiving high dose (|r| = 0.651-0.975); in the superoinferior and vector directions, breast displacement only correlated with the heart volume receiving >5 Gy (V_5_) (r = −0.795, 0.687). The lung volume and the lung volume receiving high dose correlated reasonably well (r = 0.655 ~ 0.882), and a correlation only existed between heart volume and V_5_ (r = −0.701).

**Conclusion:**

Target movement correlated well with the target/OAR dosimetric variation in certain directions, indicating that whole breast IMRT assisted by breathing control or respiratory-adapted gated treatment promotes the accuracy of dose delivery during radiotherapy. During free breathing, the effect of breast volume variation can be ignored in whole breast IMRT.

## Introduction

Breast conservation therapy is accepted as the standard treatment and the preferred treatment for early-stage breast carcinoma, and breast radiotherapy after breast-conserving lumpectomy is an important part of breast conservation therapy. Postoperative whole breast radiotherapy (WBRT) results in the reduction of local and regional recurrence and also contributes to improvements in overall survival [1]. Compared with conventional radiotherapy, intensity modulated radiotherapy (IMRT) decreases acute toxicity and allows superior cosmetic outcome [2,3], thus, IMRT is a widely accepted alternative for patients undergoing large breast-conserving surgery.

There are several factors that lead to uncertainties in target position between treatment and planning computed tomography scanning, such as setup error, breathing motion, and breast deformation. However, in recent years, with the development of sophisticated image-guided online and offline setup verification and correction techniques, such as the electric portal imaging device (EPID) and cone-beam CT (CBCT), the interfraction setup error has been significantly reduced during delivery of irradiation in breast cancer patients [4-7]. Although the intrafractional breathing motion is usually only a few millimeters [8-10], respiratory-induced movement during free breathing has recently become the focus of radiotherapy research [8,9,11,12]. A number of studies have shown that the dosimetric impact of respiratory motion was clinically insignificant during free breathing [8,9]. However, several investigations reported that the target motion influenced the dose distribution, and breathing control reduced the uncertainty in the target movement resulting from respiration [11,12]. Various techniques for postoperative WBRT have been used in different studies. In our study, the tangential field technique with static multileaf collimator segments (SMLC) IMRT was used after breast-conserving surgery.

Currently, there are few studies using four dimensional computed tomography (4DCT) both to analyze the respiration-induced target movement and volume change during WBRT, and to correlate these with the resulting dosimetric impact. The main purposes of this study were (1) to investigate the uncertainty of the treated breast motion and volume variation induced by respiration during free breathing using 4DCT, (2) to study the resulting dosimetric impact during the whole breathing cycle, and (3) correlate the magnitude of respiration-induced (1) with (2). On the basis of this correlation, we propose the treatment phases for respiratory-gated radiation therapy to achieve a high dose conformity and homogeneity around the target while sparing normal tissues.

## Methods and materials

### Patient selection and instruction

Seventeen early-stage breast cancer patients who had undergone breast-conserving surgery and were potentially eligible for adjuvant WBRT were enrolled in this study. Patients with restricted arm movements after surgery and poor pulmonary function were excluded. Ten of these 17 patients had right-sided breast cancer, and the remaining seven had left-sided breast cancer. Written informed consent was obtained from all patients and this study was approved by the institutional research ethics board of Shandong Cancer Hospital.

### 4DCT data acquisition

Patients were immobilized in the supine position on a breast board with both arms raised over the head and positioned on the arm support device. A knee support was placed under the knees to fix the position and improve patient comfort.

The free breathing 4DCT scans were acquired on a 16-slice CT scanner (Philips Brilliance Bores CT, Netherlands). Three laser alignment lines were marked on the patient before CT acquisition. The respiratory signal was recorded with the Varian real-time position management (RPM) System (Varian Medical Systems, Palo Alto, CA, USA), by measuring the displacement of the infrared markers placed on the epigastric region of the patient’s abdomen. GE Advantage 4D software (GE Healthcare, Waukesha, WI, USA) was used to sort the reconstructed 4DCT images into ten respiratory phases labeled as 0-90% on the basis of triggered signals. Phase 0% denotes the maximum end inspiration (EI) and phase 50% denotes the maximum end expiration (EE). The 4DCT images were reconstructed using a thickness of 2 mm and then transferred to the Eclipse treatment planning system (TPS) (Eclipse 8.6, Varian Medical Systems, Palo Alto, CA, USA) for structure delineation and treatment planning generation.

### Treatment planning and dosimetric evaluation

The treated breast (CTV), ipsilateral lung (IPSL), and heart were delineated on the 10 phases of the 4DCT data sets. CTV was defined based on the visible glandular breast tissue seen on the CT images, using anatomic references. In addition, the maximal displacements in the lateral (LR), anteroposterior (AP) and superoinferior (SI) directions were derived from the positions of the centre of the mass. The planning target volume (PTV) was generated using a 5 mm margin around the CTV and shrunk by 5 mm below the skin surface.

Treatment planning was established at the EI phase, using the tangential field technique with static multileaf collimator segments (SMLC) IMRT irradiating the whole breast. The prescription dose was 50 Gy at 2 Gy per fraction to the PTV using 6 MV photon beams. The criteria of SMLC-IMRT planning were to ensure at least 95% of the PTV received the prescription dose, the segmented MLCs were manipulated to shield the areas of the PTV receiving doses > 103% of the prescription dose. The dose delivered to organs at risk, such as the IPSL and heart were maintained within normal accepted tolerances. The volume of the ipsilateral lung receiving 20 Gy was <25%, and the volume of the heart receiving 30 Gy was <10%.

Treatment planning was copied and applied to each phase of the 4DCT scan, with the gantry angles, monitor units delivered per beam, collimator angles, and primary field size exactly the same for all respiratory phases. The dose distribution was calculated separately in all phases. CTV spatial motion vector $\left(\left|\vec{v}\right|\right)$ was calculated as

$$|\vec{v}|=\sqrt{LR^{2}+AP^{2}+SI^{2},}$$

in which LR, AP, SI represent the CTV displacements in the lateral, anteroposterior and superoinferior directions. For the PTV, the following parameters were evaluated: the mean dose (D_mean_), homogeneity index (HI), and conformal index (CI). HI was defined as

$$\mathrm{HI}=\frac{D_{2}-D_{98}}{\mathrm{DT}},$$

in which D_2_ and D_98_ represent the dose to 2% and 98% of the target volume, respectively, and D_T_ is the prescription dose [13]. CI was defined as

$$\mathrm{CI}=\frac{\mathrm{PT}V_{\mathrm{ref}}}{V_{\mathrm{PTV}}}\times \frac{\mathrm{PT}V_{\mathrm{ref}}}{V_{\mathrm{ref}}},$$

where PTV_ref_ represents the volume of PTV that is covered by the reference dose, V_PTV_ is defined as the planning target volume and V_ref_ represents the volume enclosed by the prescribed isodose [14,15]. The volumes receiving more than 5, 10, 20, 30, 40, 50 Gy (V_5_, V_10_, V_20_, V_30_, V_40_, V_50_) and D_mean_ were acquired for the IPSL and heart.

### Statistical analysis

Statistical analysis was performed using the SPSS statistical analysis software package. The Pearson correlation test was used to study the relationship between the motion and volume variation in CTV and PTV/OARs parameters during free breathing. We regarded a *p* value <0.05 as statistically significant.

## Results

### Target motion and volume change

In a comparison of the different 4DCT phases, the treated breast centroid movement reached 1.03 ± 0.48 mm, 0.95 ± 0.36 mm, and 1.38 ± 0.85 mm in the LR, AP, and SI directions, respectively. The CTV showed the greater movement in the SI direction compared with the LR and AP directions. The spatial motion vector was 2.09 ± 0.74 mm. Therefore, the relatively large intrafraction motion was scattered asymmetrically. The CTV volume variation during the whole breathing cycle was 3.05 ± 0.94% (generally within 1.59% - 4.91%).

### Influence of breast motion on dose

During free breathing, the homogeneity index (HI) of PTV variation was a median of 2.02% (range 0.68% - 9.79%), and the correlation between breast motion and HI revealed no statistical significance (Table 1). While the variation in conformal index (CI) during a free breathing cycle was a median of 2.60% (range 0.79% - 10.84%), statistically significant correlations were found between the breast spatial motion and CI, except in the LR direction (Table 1). The mean PTV dose, HI, and CI did not show a statistically significant correlation with the treated breast volume variance during free breathing.

**Table 1:** Correlation between the whole respiratory cycle target motion/volume and PTV and IPSL parameters

		**PTV**			**IPSL**						
		**D**_**mean**_	**HI**	**CI**	**V**_**5**_	**V**_**10**_	**V**_**20**_	**V**_**30**_	**V**_**40**_	**V**_**50**_	**D**_**mean**_
LR	*r*	−0.471	0.289	0.085	0.607	0.453	0.297	0.161	0.125	0.067	−0.307
	*P*	0.170	0.419	0.814	0.062	0.188	0.405	0.656	0.731	0.854	0.301
AP	*r*	−0.855	0.594	−0.820	−0.099	−0.653	−0.857	−0.897	−0.951	−0.975	0.798
	*P*	0.002	0.070	0.003	0.786	0.041	0.002	0.000	0.000	0.000	0.006
SI	*r*	0.693	−0.545	0.791	0.086	0.575	0.752	0.777	0.862	0.884	0.804
	*P*	0.026	0.104	0.006	0.812	0.082	0.012	0.008	0.001	0.001	0.005
Vector	*r*	−0.651	0.518	−0.846	−0.161	−0.651	−0.808	−0.824	−0.891	−0.905	−0.779
	*P*	0.041	0.125	0.002	0.657	0.042	0.005	0.003	0.001	0.000	0.008
volume	*r*	−0.200	0.250	0.316	0.459	0.455	0.416	0.346	0.352	0.354	0.480
	*P*	0.579	0.485	0.374	0.182	0.186	0.232	0.327	0.319	0.315	0.161

Abbreviations: *PTV* planning target volume; *IPSL* ipsilateral lung; *LR* lateral; *AP* anteroposterior; *SI* superoinferior; *V*_*x*_ volume receiving more than x Gy; *D*_*mean*_ the mean dose.

Table 1 shows the correlation between the treated breast movement and the lung dose-volume histograms (DVH) parameters during the respiration cycle. The volumes of the IPSL receiving more than 20 Gy (V_20_, V_30_, V_40_, V_50_) and the D_mean_ all correlated well with the treated breast motion in the AP and SI directions, and a statistically significant inverse correlation was found for the motion vector. This meant that SMLC-IMRT was sensitive to breast movement induced by breathing motion. There were no meaningful correlations between the dosimetric parameters and changes in the breast volume. In other words, during radiation therapy, breast motion has a significant effect on the IPSL dosimetric parameters rather than the breast volume change.

In the 7 left-sided breast cancer patients, the correlation coefficients between the dosimetric parameters of the heart and breast motion, and the changes in breast volume are shown in Table 2. As shown in Table 2, only V_5_ showed a good correlation with motion in the AP direction and the motion vector throughout the respiratory cycle. The heart has its intrinsic rhythm and out of sync with the breathing motion.

**Table 2:** Correlation between the whole respiratory cycle target motion/volume and heart parameters for left-sided patients

		**V**_**5**_	**V**_**10**_	**V**_**20**_	**V**_**30**_	**V**_**40**_	**V**_**50**_	**D**_**mean**_
LR	*r*	0.170	0.326	0.394	0.412	0.481	0.274	0.393
	*P*	0.638	0.358	0.236	0.236	0.159	0.444	0.377
AP	*r*	0.523	0.356	0.274	0.296	0.188	−0.397	−0.338
	*P*	0.121	0.313	0.444	0.406	0.603	0.256	0.339
SI	*r*	−0.795	−0.590	−0.44	−0.446	−0.286	0.483	0.496
	*P*	0.006	0.073	0.203	0.196	0.424	0.157	0.145
Vector	*r*	0.687	0.470	0.324	0.327	0.174	−0.509	−0.445
	*P*	0.028	0.170	0.361	0.356	0.632	0.133	0.198
volume	*r*	−0.390	−0.287	−0.196	−0.166	−0.104	0.252	−0.236
	*P*	0.265	0.421	0.588	0.647	0.775	0.482	0.512

Abbreviations: *LR* lateral; *AP* anteroposterior; *SI* superoinferior; *V*_*x*_ volume receiving more than x Gy; *D*_*mean*_ the mean dose.

### Influence of OAR volume change on dose

**Figure 1:**
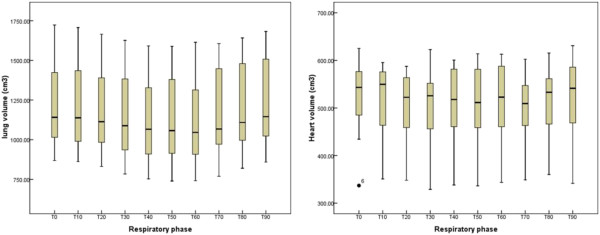
The changes in the ipslateral lung (left) and heart volume (right) in successive 4DCT phases during free breathing.

From Figure 1, it can be seen that the lung volume variation fell within the respiratory rationale, while an irregularity in the respiratory cycle led to changes in the heart volume: an uneven curve instead of a sine curve. The results of Figure 1 shows the principle of a 4DCT: the lung volume changes as a function of the breathing phase. The heart movement is much faster than the respiration triggered CT. So the change of the heart volume cannot be assessed with a respiration gated CT. Table 1 shows that the changes in the ipsilateral lung volume correlated well with the lung volume receiving more than 20, 30, 40, 50 Gy (V_20_, V_30_, V_40_, V_50_) (r = 0.655, 0.728, 0.822, 0.882). The evaluation of DVHs for the heart was performed in 7 patients with left-sided breast cancer. Preliminary data from these patients indicated that the heart volume change only correlated with the heart volume receiving more than 5 Gy (V_5_) (*r* = −0.701, Table 2).

## Discussion

Many studies have been performed using various techniques to investigate the treated breast movement by implementing respiratory management methods in radiation delivery such as EPID and 4D medical imaging [9,16]. Differences were observed to exist between these different methods. In our investigation, we concluded that peak-to-peak respiration induced the treated breast to move in the SI direction and reached a maximal value of 1.38 ± 0.85 mm, and the motion vector was 2.09 ± 0.74 mm. Other authors also investigated the motion characteristics of the treated breast using 4DCT and showed similar results. For example, Qi et al. [9] demonstrated that the maximum centroid movement ranged from 1.1-3.9 mm for the ipsilateral breast during free breathing. The motion extent measured in our study was also consistent with Richter et al. [8], who measured the motion amplitude of the breast motion in the 4D CT images. They reported that the motion amplitude of the breast motion was between 0.2 mm and 3.8 mm, 1.8 mm on average. Strydhorst et al. [17] reported that for breast, the average maximum motion of the external contour was 1.3 ± 1.6 mm. Thus, our data are consistent with the results of their study. Our study addressed whether this small intrafraction motion amplitude of the treated breast resulted in a significant variance in the dose delivered to the PTV and OARs. And intratreatment target volume variation was also investigated throughout the whole respiratory cycle.

During the respiratory cycle, the variation in the HI showed little dependence on the target displacement, while the patients breathed freely. This parameter did not correlate well with changes in the treated breast volume. However, the variance in the HI among all 17 patients studied ranged from 0.68% to 9.79%, which indicated that the deterioration in dose distributions induced by respiratory motion varied substantially between and within patients. Patients were immobilized in the supine position on a sloping breast board. As a non-rigid tissue, the breast was deformed due to the effects of gravity and breathing patterns, and the three dimensional movement correlations were asymmetrical. Thus, throughout the respiratory cycle a statistically significant inverse correlation was found between the motion vector and the mean PTV dose and CI, while there was no correlation with breast motion in the LR directions. The maximum variance of the CI reached 10.84% in our study, thus, respiration-induced breast motion resulted in altered dose distributions, and was associated with a small amplitude in target motion bearing a higher risk for lower PTV coverage. This finding indicated that although the motion amplitude of the treated breast in the 4DCT studies was small, breathing control and real time tumor tracking might improve target coverage for whole breast irradiation and have the potential to decrease the PTV dose heterogeneity.

The anatomical movement within the planned radiation field induced by breathing motion can lead to a decrease in target coverage and normal tissue sparing, resulting in an increased risk of treatment failure and normal tissue complication probability (NTCP) [18]. Acute lung pneumonia and late lung fibrosis depends on the lung volume irradiated during WBRT [19,20]. Analysis of the treated breast movement and the volume variation of the ipsilateral lung for these 17 patients showed a strong correlation with the high dose delivered to the lung during the respiratory cycle. The findings in our study confirmed that the deterioration in lung dose during free breathing were also relatively insensitive to breast motion and thorax expansion. This was consistent with a study by Cao et al. [21], where a linear model was developed to relate the dosimetric coverage difference introduced by respiration with the motion information. Based on their studies, they recommend that beamlet IMRT with motion of a medial marker >0.6 cm is not used for whole-breast radiotherapy unless the use of respiration control is considered. We further researched the relationship between lung volume variation and the dosimetric coverage. And found that during the respiratory cycle, patients with smaller lung volume in end expiration phases were associated with decreased dose to the IPSL. Hence, for WBRT, breathing control or respiratory-adapted gated treatment may spare the IPSL compared with the conventional non-gated plan in a subset of patients with large lung volume variations, and respiration gating may be more precise if performed at the EE phase. From the results of these 17 patients, it is not possible to propose guidelines for the selection of patients suitable for advanced techniques such as active breathing control (ABC) and real time tumor tracking. However, we suggest that breathing control, real time tumor tracking and image-guided radiotherapy may be clinically significant for whole breast IMRT in addition to what was achieved by the technique described here.

Radiotherapy for left breast cancer is associated with an increased risk of acute or chronic cardiotoxicity, such as ischemic heart disease, pericarditis, and myocardial infarction, long after treatment. The data from Figure 1 shows that the heart volume variation did not follow the respiratory patterns during a normal free breathing cycle, resulting in a higher likelihood of intrinsic cardiac contractions. The technique of SMLC-IMRT combined with the limited heart volume included in the radiation fields, showed that breast motion did not significantly correlate with dose distribution within the heart during WBRT. Using 4DCT images, Qi et al. [12] demonstrated that the distance from the left ascending aorta to a fixed line (DLAD) and the maximum heart depth (MHD) changed up to 9 and 11 mm, respectively, and were positively correlated with mean heart dose and heart dose volume. The differences between the results in our study and Qi’s investigation might be due to differences in radiotherapy technique. We treated the whole breast with SMLC-IMRT, while Qi et al. used conventional 3D planning with tangential beams for whole breast and a boost to the lumpectomy bed PTV. Although the respiration-induced breast movements played only a minimal part in the heart dosimetric variance, heart movement should be used independently as a consideration for the respiratory gating procedure before treatment planning. Hence, respiratory-adapted gated treatment or breathing control may spare the heart compared with the conventional plan.

Our study focused on the analysis of breathing motion and volume change, target delineation errors were not considered. However, this uncertainty may affect the accuracy of the dose distribution and dose homogeneity in the treated breast, therefore, all targets and OARs should be consistently delineated by one radiotherapist using uniform criteria to decrease geometrical uncertainties [22]. During the respiratory cycle, mean breast volume change was small (<4%), and the correlations with PTV and OARs dose variation were not significant. Our work has shown that intrafractional changes in the breast volume were generally clinically insignificant and did not greatly influence the dose distribution, therefore can be ignored during radiotherapy.

## Conclusion

The treated breast volume change during respiration can be ignored for whole breast forward intensity modulated radiotherapy during free breathing. The respiratory-induced motion of the breast is usually only a few millimeters, but may also decrease target coverage and the resulting dosimetric impact. This indicates that small breathing motions can lead to a change in the dose distributions, and 4D treatment planning and/or breathing control for tangential field breast radiotherapy should improve the accuracy of dose delivery.

## Abbreviations

4DCT: Four-dimensional computed tomography; ABC: Active breathing control; AP: Anteroposterior; CBCT: Cone-beam computed tomography; CI: Conformal index; EE: End-expiration; EI: End-inspiration; EPID: Electric portal imaging device; HI: Homogeneity index; IMRT: Intensity-modulated radiotherapy; IPSL: Ipsilateral lung; LR: Lateral; NTCP: Normal tissue complication probability; OAR: Organ at risk; PTV: Planning target volume; RPM: Real-time Positioning Management; SI: Superoinferior; SMLC-IMRT: Static multileaf collimator segments IMRT; TPS: Treatment planning system; WBRT: Whole breast radiotherapy.

## Competing interests

The authors declare that they have no competing interests.

## Authors’ contributions

WW, LJB participated in the study design, contributed to the data collection, and draft the manuscript. HHG, ST, LJ participated in the treatment planning. LFX, XM made important contributions in collecting and analyzing data, and in revising the content. All authors read and approved the final manuscript.
